# Dependence of Photoresponsivity and On/Off Ratio on Quantum Dot Density in Quantum Dot Sensitized MoS_2_ Photodetector

**DOI:** 10.3390/nano10091828

**Published:** 2020-09-14

**Authors:** Yung-Yu Lai, Yen-Wei Yeh, An-Jye Tzou, Yi-Yuan Chen, YewChung Sermon Wu, Yuh-Jen Cheng, Hao-Chung Kuo

**Affiliations:** 1 Department of Materials Science and Engineering, National Chiao Tung University, Hsinchu 30010, Taiwan; loveriver031@gmail.com (Y.-Y.L.); sermonwu@faculty.nctu.edu.tw (Y.S.W.); 2 Research Center for Applied Sciences, Academia Sinica, Taipei 11529, Taiwan; fredyeh.eo06g@g2.nctu.edu.tw; 3 Department of Photonics and Institute of Electro-Optical Engineering, College of Electrical and Computer Engineering, National Chiao Tung University, Hsinchu 30010, Taiwan; yoyo91576@gmail.com; 4 Taiwan Semiconductor Research Institute, National Applied Research Laboratories, Hsinchu 30078, Taiwan; ajtzou@narlabs.org.tw

**Keywords:** molybdenum disulfide, quantum dots, photodetectors, non-radiative energy transfer

## Abstract

Non-radiative energy transfer (NRET) from quantum dots (QDs) to monolayer MoS_2_ has been shown to greatly enhance the photoresponsivity of the MoS_2_ photodetector, lifting the limitations imposed by monolayer absorption thickness. Studies were often performed on a photodetector with a channel length of only a few μm and an active area of a few μm^2^. Here, we demonstrate a QD sensitized monolayer MoS_2_ photodetector with a large channel length of 40 μm and an active area of 0.13 mm^2^. The QD sensitizing coating greatly enhances photoresponsivity by 14-fold at 1.3 μW illumination power, as compared with a plain monolayer MoS_2_ photodetector without QD coating. The photoresponsivity enhancement increases as QD coating density increases. However, QD coating also causes dark current to increase due to charge doping from QD on MoS_2_. At low QD density, the increase of photocurrent is much larger than the increase of dark current, resulting in a significant enhancement of the signal on/off ratio. As QD density increases, the increase of photocurrent becomes slower than the increase of dark current. As a result, photoresponsivity increases, but the on/off ratio decreases. This inverse dependence on QD density is an important factor to consider in the QD sensitized photodetector design.

## 1. Introduction

Applications of two-dimensional (2D) semiconductor materials in optoelectronics have attracted great research interest due to their unique atomically thin profile, mechanical flexibility, and potential high electron mobility and gain [1,2,3,4,5]. Graphene for example, has gained intense attention for its extremely high mobility and fast photo response [6,7]. However, the lack of bandgap in graphene leads to high dark currents, and therefore a low on/off ratio, which limits its applications for active semiconducting channels in optoelectronic devices. Interestingly, a new emerging 2D transition metal dichalcogenides (TMDs), such as MoS_2_, has a sizable bandgap and change from an indirect bandgap material of a few layer thicknesses to a direct bandgap material of monolayer thickness [8,9]. The open bandgap is advantageous for device applications that require low dark currents to achieve a high on–off ratio [10,11]. Multilayer and monolayer MoS_2_ photodetectors and transistors have gained great research interests in recent years [12,13,14,15,16,17,18]. Multilayer MoS_2_ has a greater thickness to absorb incident light, but the indirect bandgap property leads to a lower absorption coefficient, which compromises overall photo response. Monolayer MoS_2_, in contrast, has a direct bandgap with a large absorption coefficient, but the monolayer thickness limits the absorption of incident light.

An attractive method to overcome this thickness-limited absorption is to add a sensitizing material on top of the 2D material. There have been a few reports of dye sensitized [19] and quantum dot (QD) sensitized MoS_2_ photodetectors [20,21,22,23,24,25,26]. QDs in particular have the advantages of high quantum yield, broadband absorption, and tunable absorption wavelength by changing its size or composition. Light incident on QDs excites excitons, which are then converted to excitons in 2D material through near field dipole-dipole interaction when QDs and 2D material are in close proximity. This non-radiative energy transfer (NRET) is very efficient when the emission spectrum of QD matches with the absorption spectrum of 2D material [27,28,29,30,31]. The excited excitons are dissociated into free charge carriers by the electric field applied between the source and drain metal contacts on MoS_2_ detector. Photo induced charges in QDs may also transfer to MoS_2_ through tunneling. This charge transfer (CT) can contribute to photocurrent as well, but it is limited to a much shorter separation < 2 nm and often leads to a lower contribution. QD sensitized 2D material photodetectors have been demonstrated on mechanically exfoliated or chemical-vapor-deposition (CVD) grown MoS_2_. A photoresponsivity of 21 mA/W and 8- to 14-fold photocurrent enhancement has been reported from using CdSeS/ZnS QDs on CVD grown MoS_2_ at 1 μW illumination power [23,24]. Photoresponsivity of 100 A/W with 16-fold photocurrent enhancement has also been reported from using ZnCdSe/ZnS QDs on exfoliated MoS_2_ at 1 μW light power [25]. The much larger responsivity of the latter report is due to the better crystalline quality of exfoliated MoS_2_ and the large charge doping from QDs. In these reports, the source and drain electrodes are often separated by a channel length of a just few μm under 1 V bias voltage with active area of just a few μm^2^. Here, we report CdSe/ZnS core/shell QD sensitized CVD monolayer MoS_2_ photodetectors using interdigital electrodes with a large channel length of 40 μm. Despite the possibility of encountering more surface defects and grain boundaries due to large channel length, which may cause transport scattering and non-radiative carrier recombination, the photocurrent is still enhanced up to 14 times by the sensitizing QDs at 1.3 μW illumination power. Another important performance factor of photodetector is the signal on/off ratio. Adding QD coating can greatly enhance photocurrent. Concomitantly, it also changes the charge doping of MoS_2_ and affects dark current. We find that both photocurrent and dark current increases with QD coating density. At low QD density, the photocurrent to dark current (on/off) ratio is greatly enhanced. As QD density increases, photocurrent however does not increase as fast as dark current. As a result, the enhancement decreases as QD density increases, even though the on/off ratio is still enhanced as compared with that of the MoS_2_ photodector without QD coating. This decrease of on/off ratio with QD density can set an upper limit on the QD coating density.

## 2. Materials and Methods

**Figure 1:**
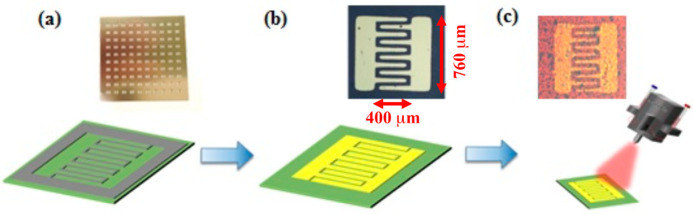
Photodetector fabrication process. (**a**) Physical mask on sample. (**b**) Deposition of Ti/Au (10/50 nm) on sample. (**c**) Spray coating of CdSe/ZnS QDs.

The MoS_2_ film was grown on a c-plane sapphire substrate by chemical vapor deposition (CVD) using MoO_3_ and sulfur powder as precursors in a quartz tube furnace [22]. MoS_2_ photodetectors were fabricated using a physical stencil mask to pattern interdigital electrodes on a MoS_2_ film, as shown in Figure 1a. The interdigital fingers have a spacing of 40 µm. There are five finger pairs and each finger length is 400 µm. The physical mask was placed on top of a MoS_2_ film on a sapphire substrate. E-gun deposition was used to deposit Ti (10 nm) and Au (50 nm) on the substrate to form source-drain metal contact electrodes on MoS_2_ (Figure 1b). The physical mask was then removed and core/shell CdSe/ZnS QDs (UT Dots, CSZ630) diluted in Toluene (C_7_H_8_) solution with volume ratio of 1:100 were sprayed on the device by a nano-particle pulsed-spray coater (Hermes-Epitek, Singapore, Singapore). One spray pulse produces a QD coating density of about 1.8 × 10^11^ QDs/cm^2^. The CdSe/ZnS QD has a diameter of ~6 nm and emission peak wavelength at 630 nm. QDs were deposited on the whole sample surface area, but only those on the active region between electrode fingers contributed to the photo current enhancement. The light intensity integrated over the active area between interdigital electrodes was used to report the illumination power throughout the paper. The fluorescent optical microscope image of the fabricated QD sensitized monolayer MoS_2_ photodetector is shown in Figure 1c. The MoS_2_ material quality and layer thickness were verified using photoluminescent measurement and Raman spectroscopy. Samples with different numbers of repeating QD sprays were fabricated to study the dependence of photocurrent on QD density. Photoresponsivity was measured under different illumination intensity, source-drain biased voltage, and QD density. The response time of MoS_2_ photodetector was measured using a laser with fast on-off power modulation. The efficiency of NRET from QD to MoS_2_ was investigated using time resolved photoluminescent measurement.

## 3. Results and Discussions

**Figure 2:**
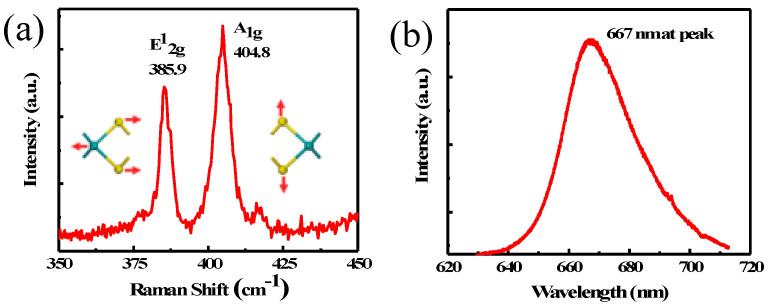
(**a**) Raman spectrum. (**b**) PL spectrum of MoS_2_ film.

Raman spectrum measurement provides a convenient and nondestructive method to measure the layer number of 2D materials [32,33]. There are two Raman peaks corresponding to in-plane vibration mode E^1^_2g_ and out-of-plane vibration mode A_1g_ of MoS_2_, respectively. The separation between these two peaks depends on layer number. The measured Raman spectrum shows characteristic peaks of MoS_2_ with in-plane mode E^1^_2g_ at 385.9 cm^−1^ and out-of-plane mode A_1g_ at 404.8 cm^−1^, as shown in Figure 2a. The difference between these two modes is 18.9 cm^−1^, which indicates a monolayer MoS_2_ [33]. Photoluminescence (PL) is another useful non-destructive method to evaluate the crystal quality of MoS_2_ film. The principle of PL measurement involves using a laser with photon energy greater than the semiconductor energy bandgap to excite electron–hole pairs in the sample. The excited electron–hole pairs relax to band edge and recombine to emit photons when the material is in good quality. If there are many defects in the material, the emission will be broad and weak due to loss to non-radiative recombination at defects. Figure 2b shows the measured PL spectrum with a distinct emission peak at 667.5 nm, corresponding to a bandgap of ~1.86 eV. The PL spectral linewidth is ~28 nm, which indicates a reasonably good material quality [25].

**Figure 3:**
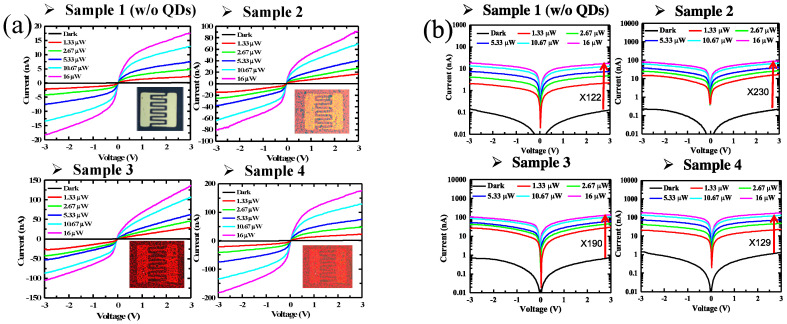
*I_ds_*–*V_ds_* curves of MoS_2_ monolayer photodetectors without QD coating in linear (**a**) and log scale (**b**).

We have fabricated four MoS_2_ photodetector samples to study the dependence of photoresponse on QD coating density. These include samples without QDs and having 10, 15, and 20 applications of pulsed-spray QD coating, referred as Sample 1 to Sample 4, respectively. The corresponding QD density increases from Sample 2 to 4 with QD density of 1.8 × 10^12^, 2.7 × 10^12^, and 3.6 × 10^12^ QD/cm^2^. The photoresponses of these samples were characterized by measuring photocurrent *I_ds_* versus bias voltage *V_ds_* applied across the interdigital source-drain electrodes under different illumination power. The illumination source is a laser with emission wavelength at 450 nm. The measured *I_ds_*–*V_ds_* curves in linear scale are shown in Figure 3a. Each sample was measured under different illumination power ranging up to 16 μW. Comparing among samples 1 to 4 (note the different y-axis scale in each figure), photocurrent *I_ds_* increases as QD density increases. Under 16 μW illumination power and 3 V bias voltage, the photocurrents of Sample 2 to 4 are respectively enhanced by 5.3, 8.1, and 11 times, as compared with that of Sample 1 without QD coating. The large photocurrent enhancement demonstrates that QDs are effective in absorbing incident photons and converting them to conducting charge carriers. The *I_ds_*–*V_ds_* curves are symmetric, but have a nonlinear dependence on bias voltage *V_ds_*, which will be discussed shortly. A log scale graph is shown in Figure 3b to show dark current. Comparing the dark currents from Sample 1 to 4, we see the dark current increases as QD coating density increases. QD coating introduces charge doping to MoS_2_ and increases carrier density. This increase in dark current has a negative effect on the photocurrent on/off ratio. Comparing among Sample 1 to 4 under 16 μW illumination power and 3 V bias voltage, the on/off ratio increases from 121 for Sample 1 (without QD) to 230 for Sample 2 (with QD), then decreases to 190 and 129 for Sample 3 and 4, respectively. These results show that QD coating can enhance both photocurrent and on/off ratio. As QD density increases, dark current has a faster increasing rate than photocurrent. As a result, the on/off ratio decreases from Sample 2 to 4. From this decreasing trend, the on/off ratio can potentially become lower than that of the pristine MoS_2_ photodetector Sample 1 if QD coating density is too high. These results show that QD coating is a very effective method for sensitizing the MoS_2_ photodetector. However, the on/off ratio will be compromised if QD density becomes too high, which is an important factor to consider when optimizing QD density for photodetector design.

**Figure 4:**
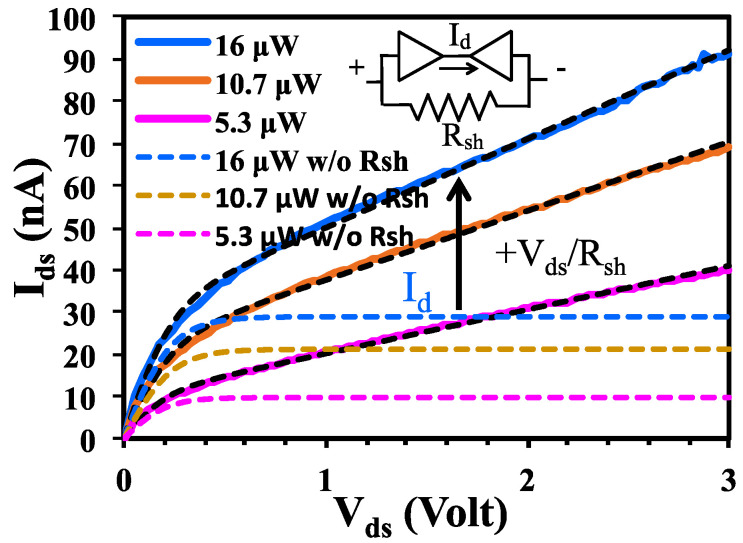
*I_ds_*–*V_ds_* curves (solid color lines) of Sample 2 and the fitted *I_ds_*–*V_ds_* curves (black dotted lines) under different illumination intensity using the circuit model shown in the inset. Current flowing through diode from curve fitting are plotted in dotted color lines.

We now study the nonlinear dependence of *I_ds_* on *V_ds_* using a Schottky junction model. The change from initial fast increase to a slow increase in *I_ds_*–*V_ds_* curve resembles the *I*–*V* characteristic of two back-to-back Schottky junctions [34,35,36]. Theoretically, *I_ds_* is limited by the reverse saturation current of Schottky junction when *V_ds_* is large. In a more advanced theory, the saturation current depends on the exponential of the fourth root of *V_ds_* by image force Schottky barrier lowering or square root of *V_ds_* by electrostatic doping [37,38]. Interestingly, we observe a linear increase of *I_ds_* in the *V_ds_* > 0.5-volt range. This does not fit to the exponential dependence on the fourth or square root of *V_ds_*. To describe this linear dependence on *V_ds_*, we introduce a shunt resistance *R_sh_* in parallel to a balk-to-back Schottky junction circuit model, as shown in the inset of Figure 4. This resistance decreases as illumination intensity increases, as indicated by the increasing *I_ds_*/*V_ds_* slope in Figure 3a when illumination intensity increases. Using standard Schottky diode equation, the voltage drop across the back-to-back Schottky junctions, one under forward *V_F_* and the other reverse *V_R_* bias, are

(1) $$V_{F}=\;-\frac{nkT}{e}\ln\left(1-\frac{I_{d}}{I_{0}}\right)\;\mathrm{and}\;V_{R}=\;\frac{nkT}{e}\ln\left(1+\frac{I_{d}}{I_{0}}\right)$$

where *e* is the electron charge, *k* is the Boltzmann constant, *T* is the absolute temperature, and *n* is the ideality factor. $I_{d}$ is the intrinsic diode current (excluding photocurrent). The reverse saturation current $I_{0}$ depends on Schottky barrier height ϕ by $I_{0}=AA^{*}T^{2}\exp\left(-e\phi /kT\right)$, where *A* is the channel area and A* is the Richardson constant. Here, we assume Schottky barrier ϕ for two Schottky contacts are the same. The overall voltage drop across the back-to-back diodes is $V_{ds}=\;V_{F}+V_{R}$. Substituting Equation (1) for *V_F_* and *V_R_*, we can express *I_d_* in terms of *V_ds_*. Adding shunt resistance current *V_ds_*/*R_sh_* to *I_d_*, we obtain the total current *I_ds_*,

(2) $$I_{ds}=I_{0}\frac{\exp\left(\frac{e}{nkT}V_{ds\;}\right)-1}{\exp\left(\frac{e}{nkT}V_{ds\;}\right)+1}+\;\frac{V_{ds}}{R_{sh}}\;$$

The measured *I_ds_*–*V_ds_* curves in Figure 3 are fitted using this simplified circuit model with *I_0_*, *R_sh_*, and *n* as fitting parameters. Typical fitting curves (black dotted lines) to the experimental *I_ds_*–*V_ds_* data from Sample 2 (solid color lines) are shown in Figure 4. This circuit model shows excellent fitting to all experimental data. The fitted parameters (*I_0_*, *R_sh_*, *n*) for 16, 10.7, and 5.3 μW intensity curves of Sample 2 are (28.9 nA, 47 MΩ, 4.7), (21.1 nA, 61 MΩ, 4.6), and (9.7 nA, 96 MΩ, 4.2), respectively. The fitted ideality factors are in the 4.7 to 4.2 ranges. Diode current *I_d_* (first term in Equation (2)) obtained from fitting is also plotted in Figure 4 (dotted color lines), which is limited by saturation current *I_0_* at *V_ds_* > 0.5 volt. Comparing *I_ds_* to *I_d_* in Figure 4, the linear increase of *I_ds_* at *V_ds_* > 0.5 volt is well described by shunt resistance current. The fitted shunt resistance *R_sh_* decreases from 96 to 47 MΩ as illumination intensity increases from 5.3 to 16 μW. The physical origin of this dependence is attributed to the increase of charge carrier density by photo excitation. The channel conductivity therefore increases, as indicated by the increase of *I_ds_*–*V_ds_* slope with illumination intensity in Figure 3. The fitted reverse saturation current *I_0_* increases with illumination intensity. Theoretically, reverse saturation current is dictated by Schottky barrier height, which depends on the equilibration among metal contact work function, semiconductor electron affinity, and Fermi level pinning by interface states. Here, the dependence of saturation current on illumination intensity indicates that photo excited charge carriers can reduce Schottky barrier height. Due to the nature of 2D material structure, the surface of monolayer 2D material presumably has less surface defect sites and dangling bonds, as compared with conventional 3D material. It therefore has weaker Fermi level pinning and interaction with metal contacts. This enables easy modification of Schottky barrier height by the photo excited carriers.

**Figure 5:**
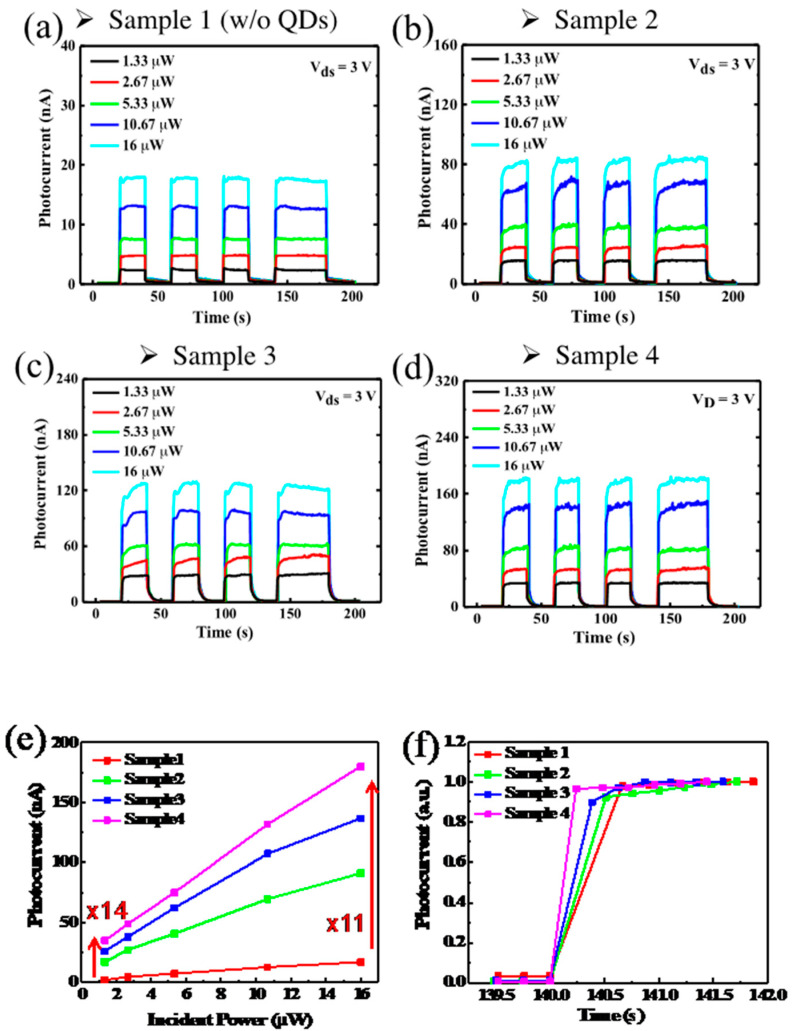
(**a**–**d**) Time-dependent photocurrent for Sample 1 to 4. (**e**) Photocurrent versus incident power at *V_ds_* = 3 V. (**f**) Rise time for Sample 1 to 4: 0.68, 0.51, 0.39, and 0.24 s.

We then measured the time-dependent photocurrent response at *V_ds_* = 3 V under different illumination power. The transient responses of photocurrents are shown in Figure 5a–d. The photocurrent response is clearly enhanced by QD coating. Taking 16 μW illumination power for example, the photocurrent increases from 17 nA for Sample 1 without QDs, to 90, 135, and 180 nA for Sample 2 to 4 with increasing QD density. The change of photocurrent as a function of illumination power for four samples is shown in Figure 5e. Photocurrent for Sample 4, as compared with Sample 1 without QDs, is enhanced by 11 times. It is interesting to note that the enhancement increases to 14 times when illumination power decreases to 1.3 μW. This clearly indicates a very efficient energy transfer by QDs to convert incident photons into charge carriers. Figure 5f shows the normalized turn-on photocurrent response curves to show the change in rise time by different QD density. The photocurrent rise time changes from 0.68 for Sample 1 without QDs to 0.51, 0.39, and 0.24 s for Sample 2 to 4 with QDs. This indicates that the QD assisted energy conversion improves response time. It is worth noting that there is a much longer response time of ~15 s with a small amplitude increase after the initial fast sub sec photocurrent rise in Figure 5a–d. This long response time is attributed to the capture or release of charge carries by impurities or defect states in MoS_2_, which can be further improved by surface passivation or using better crystalline MoS_2_.

**Figure 6:**
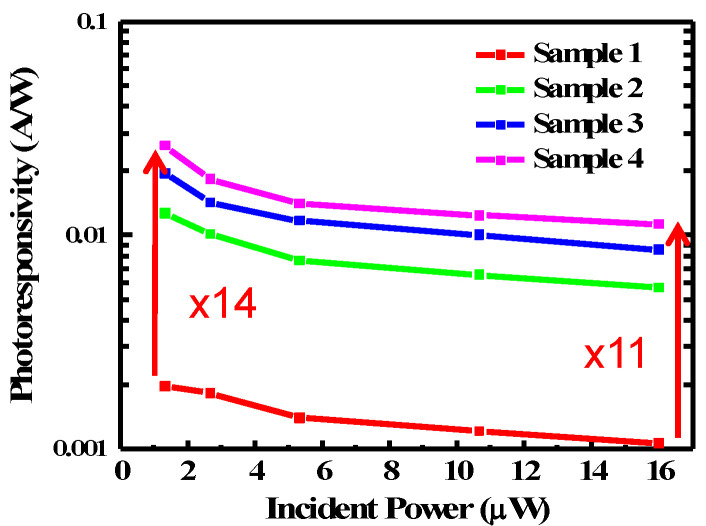
Photoresponsivity versus incident power.

From the measured photocurrent response, we obtain the photoresponsivity as a function of illumination power for all samples at *V_ds_* = 3 V, as shown in Figure 6. The photoresponsivities of Sample 1 to 4 at 16 μW illumination power are 1.0, 5.7, 8.6, and 11 mA/W, respectively. The photoresponsivity increases as QD density increases. This is expected because there are more QDs to absorb incident photons. The increasing rate, however, is slightly reduced as QD density increases. At 1.3 μW illumination power, the photoresponsivities of Sample 1 to 4 become 1.7, 12, 19, and 26 mA/W, respectively. For all samples, we see the photoresponsivity decreases as illumination power increases. This dependence could be attributed to several factors [25,39,40]. It could be due to the decrease of space charge region when illumination intensity increases. The decrease of space charge region is accompanied by a reduced internal field. This internal field is important for preventing the recombination of the photoexcited excitons. The generation rate of free charge carriers thus decreases. Heating effect and the saturation of sensitizing traps in QDs and at high illumination intensity may also result in a decrease in photoresponsivity [41,42,43]. The higher photoresponsivity at lower illumination power leads to a higher photoresponsivity enhancement. Comparing Sample 1 and 4, the photoresponsivity is enhanced by 14 times at 1.3 μW, as compared with 11 times at 16 μW illumination power.

**Figure 7:**
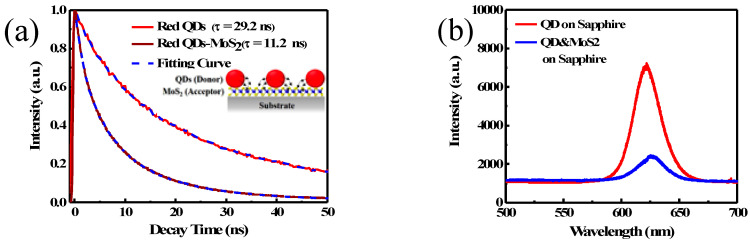
(**a**) TRPL decay curves of the QDs and QDs on MoS_2_. (**b**) PL spectrum of QDs on sapphire and QDs on MoS_2_.

Time-resolved photoluminescence (TRPL) measurement was used to study the energy transfer rate between QDs and MoS_2_. The photo excited electrons and holes in QDs may recombine through radiative and non-radiative process inside QDs. It leads to a decay in photoluminescent signal at QD emission wavelength. The decay rate of QDs on a plain sapphire substrate is $\gamma_{QD}=\gamma_{r}+\gamma_{nr}$, where $\gamma_{r}$ and $\gamma_{nr}$ are radiative and non-radiative decay rate, respectively. When QDs are coated on MoS_2_ film, the additional NRET charge transfer from QDs to MoS_2_ adds a new decay path. The new decay rate becomes $\gamma_{QD-MoS_{2}}=\gamma_{r}+\gamma_{nr}+\gamma_{NRET}$, where $\gamma_{NRET}$ is the NRET rate. The measured TRPL curves for QDs on sapphire and QDs on MoS_2_ are shown in Figure 7a. The decay curves were fitted with a biexponential fitting curve and the intensity weighted average lifetime was calculated using the fitting parameters [23,24]. The fitted decay lifetime of QDs on sapphire and QDs on MoS_2_ are $1/\gamma_{QD}$ = 29.2 and $1/\gamma_{QD-MoS_{2}}\;=$ 11.2 ns, respectively. The much faster decay rate for QDs on MoS_2_ indicates that a significant portion of excited charge carriers in QDs is transferred to MoS_2_. To further verify this transfer of charge carriers from QDs to MoS_2_, PL spectrum of QDs on a sapphire and on a MoS_2_ substrate were measured, as shown in Figure 7b. PL is significantly quenched when QDs are coated on MoS_2_, as compared with QDs directly coated on a sapphire substrate, indicating the charge carriers are lost to MoS_2_ and recombined non-radiatively. From the measured values, we obtain the NRET rate $\gamma_{NRET\;=\;}\gamma_{QD\_MoS_{2}}-\gamma_{QD}$. The NRET efficiency is defined as the ratio of the rate of NRET to the rate of total energy decay of QDs on MoS_2_, i.e., $\eta_{NRET}\;=\;\gamma_{NRET}/\gamma_{QD-MoS_{2}}$. From the measured $\gamma_{QD}$ and $\gamma_{QD-MoS_{2}}$ values, we obtain NRET efficiency $\eta_{NRET}=\;62\%$. This TRPL measurement shows that NRET is a fairly efficient process to transfer the photo-excited charge carriers from QDs to MoS_2_. This efficient NRET, together with high absorption coefficient of QDs, provides an effective way to convert the incident photons to charge carriers in MoS_2_ and enhances photoresponsivity.

## 4. Conclusions

We have studied the photocurrent response of a hybrid QD-MoS_2_ photodetector with channel length of 40 μm and active area of 0.13 mm^2^. The photoresponsivity of this photodetector is 26 mA/W under 1.3 μW incident light power and 3 V bias voltage, which is enhanced by 14 times of that of a pristine MoS_2_ photodetector. TRPL measurement indicates that a significant 62% of the energy absorbed by QDs from incident light is transferred to MoS_2_ by NRET process. Photocurrent enhancement increases from 11 to 14 times as incident light power decreases from 16 to 1.3 μW. The photoresponsivity increases from 11 to 26 mA/W accordingly. QD coating demonstrates an effective approach to enhance MoS_2_ photoresponsivity. QD coating however also increases dark current due to charge doping from QD coating. At low QD density, photocurrent increase is larger than dark current increase, leading to an enhancement in photocurrent on/off ratio. At high QD density, the photocurrent increase is not as large as dark current increase. As a result, the on/off ratio enhancement decreases as QD density increases. These studies demonstrate the potential of hybrid QD-MoS_2_ photodetector application and show the opposite dependence of photoresponsivity and on/off ratio on QD density, which are important factors to consider for photodetector design optimization.
